# Tomographic Evaluation of the Bronchial and Pulmonary Vascular Relationships in Cats Naturally Infected with Immature *Dirofilaria immitis*

**DOI:** 10.3390/ani15223320

**Published:** 2025-11-18

**Authors:** Sara Nieves García-Rodríguez, Jorge Isidoro Matos, Laín García-Guasch, Eva Mohr-Peraza, José Alberto Montoya-Alonso, Elena Carretón

**Affiliations:** 1 Internal Medicine, Faculty of Veterinary Medicine, Research Institute of Biomedical and Health Sciences (IUIBS), University of Las Palmas de Gran Canaria, 35413 Las Palmas de Gran Canaria, Spain; saranieves.garcia@ulpgc.es (S.N.G.-R.); lain.garcia@ivcevidensia.es (L.G.-G.); eva.mohr@ulpgc.es (E.M.-P.); alberto.montoya@ulpgc.es (J.A.M.-A.); elena.carreton@ulpgc.es (E.C.); 2 IVC Evidensia Hospital Veterinari Molins, 08005 Barcelona, Spain; 3 IVC Evidensia Hospital Veterinaria del Mar, 08005 Barcelona, Spain

**Keywords:** *Dirofilaria immitis*, feline heartworm disease, HARD, computed tomography, bronchoarterial ratio, bronchus-to-vein ratio, pulmonary vein-to-artery ratio

## Abstract

Feline heartworm disease (*Dirofilaria immitis)* often leads to early pulmonary lesions even in infections by immature stages. Diagnosis is challenging due to nonspecific signs and limited sensitivity of serological tests, particularly in immature infections. To evaluate the relationships between the bronchial lumen and the pulmonary vasculature, 38 cats were evaluated thought computed tomography (CT). Of them, 30 were seropositive for *D. immitis* antibodies with respiratory signs and 8 seronegative controls. Thoracic CT scans were performed, and bronchial and vascular structures were measured. Ratios analyzed included bronchus-to-artery (BA), bronchus-to-vein (BV), and pulmonary vein-to-artery (PV/PA) ratios. Statistical comparisons were conducted across groups and lung lobes. Median BA and BV ratios were significantly higher in seropositive cats across most lung lobes. PV/PA ratios did not differ significantly between groups. Differences across lobes were significant in infected cats for BA and BV ratios. These findings indicate early bronchial remodeling in naturally infected cats, whereas pulmonary arteries and veins were less affected at this stage. Thus, BA and BV ratios are sensitive indicators of early pulmonary involvement and may improve diagnostic accuracy when used alongside conventional serological and imaging tests, which could support early identification and management of feline heartworm disease in endemic regions.

## Simple Summary

Feline heartworm disease, caused by *Dirofilaria immitis*, affects both the bronchi and pulmonary arteries and can result in restrictive lung disease. Diagnosis is challenging due to nonspecific clinical signs and the limited reliability of serological and antigen tests, particularly in immature infections. Computed tomography (CT) allows detailed evaluation of pulmonary structures and may detect early lesions not visible on standard radiographs. In this study, naturally infected seropositive cats were compared with seronegative controls using bronchus-artery (BA), bronchus-vein (BV), and pulmonary vein/artery (PV/PA) ratios. Results showed that bronchi were the most affected structures, with generalized dilation in most lung lobes and significantly increased BA and BV ratios. PV/PA ratios did not differ significantly, indicating limited early vascular involvement. These findings highlight the importance of assessing bronchial structures via CT for early detection of feline heartworm disease. Quantitative imaging parameters, such as BA and BV ratios, could complement conventional diagnostic tools and improve the presumptive diagnosis of feline heartworm, especially in endemic areas.

## 1. Introduction

Feline cardiopulmonary dirofilariosis (heartworm), caused by *Dirofilaria immitis*, affects both the bronchi and pulmonary arteries, often causing lesions in the bronchial tubes and pulmonary vasculature. It may also result in restrictive lung disease due to interstitial myofibrocytic proliferation [1,2,3]. In cats, heartworm infection can occur in two forms. The first is associated with the arrival and death of immature *D. immitis* larvae, causing the so-called Heartworm-Associated Respiratory Disease [HARD], which triggers an acute inflammatory reaction in the lung and activates pulmonary intravascular macrophages [3,4]. The second form, less common, occurs when the larvae develop into adult parasites, leading to chronic feline heartworm disease [5].

In both forms, affected cats may exhibit respiratory symptoms such as coughing and/or dyspnea, mainly. These signs are frequently misdiagnosed as more common conditions, such as feline asthma and chronic bronchitis [2,6]. Many cats may also remain asymptomatic or display non-specific clinical signs [7].

The diagnosis of feline heartworm disease is often complex and typically involves a combination of diagnostic approaches, such as serological or molecular assays for antigen or DNA detection, Knott’s test for circulating microfilariae, and/or echocardiography. In many cases, a definitive diagnosis cannot be achieved, and infection is only suspected based on indirect findings, mostly radiographic changes compatible with heartworm disease and clinical signs [1]. According to several publications, computed tomography (CT) is gaining increasing importance as a diagnostic tool for thoracic diseases [8,9,10]. Despite its higher cost, CT offers greater sensitivity than radiography for detecting lesions, as radiographic images often suffer from overlapping structures that hinder visualization [11]. One of the advantages of CT is the ability to perform 3D or multiplanar reconstructions, allowing for spatial assessment and better visualization of anatomical structures. It has also been noted that several factors—such as patient characteristics, injection duration, and injection velocity—can influence contrast enhancement and thus the appearance of structures [9,12]. Moreover, CT is a valuable technique for identifying bronchiectasis and pulmonary hypertension of precapillary origin [13,14,15].

In human medicine, CT has high diagnostic value, as it enables visualization and evaluation of lung lesions and nodules associated with *D. immitis* infections [16,17]. It is also considered the gold standard for diagnosing pulmonary thromboembolism [17]. Given its demonstrated utility in humans, CT has become increasingly used in veterinary medicine to support the diagnosis of canine and feline heartworm disease by providing standardized measurements and detailed assessment of cardiopulmonary and vascular structures [2,18]. Furthermore, since the sudden death of the parasite may result in pulmonary thromboembolism and exacerbate vascular damage, the pulmonary vascular and bronchial structures are key areas of interest in this pathology [2,7,16,18,19].

Computed Tomography studies in cats infected by *D. immitis* have detected pulmonary vascular and bronchial damage. Findings consistent with restrictive lung disease have also been reported, including irregular increases in lung opacity and reduced lung volumes without hyperinflation [2]. However, studies evaluating pulmonary lesions and CT findings in naturally infected cats remain limited.

Previous research has described the anatomical relationship between the bronchus and pulmonary artery in healthy cats, highlighting its utility in identifying abnormal bronchi in infected individuals [20,21]. Other studies have examined the diameter of bronchial and pulmonary artery structures in cats experimentally infected with *D. immitis* [22]. Nevertheless, the ratios between bronchial lumen and pulmonary vein (BV), bronchial lumen to pulmonary artery (BA), and pulmonary vein to pulmonary artery (PV/PA) have not been previously evaluated in cats naturally infected with immature *D. immitis* larvae. Therefore, the aim of this study was to evaluate the BA, BV, and PV/PA ratios obtained via CT scan in a group of symptomatic, seropositive feline patients naturally infected with immature stages of *D. immitis*.

## 2. Materials and Methods

### 2.1. Animals

A total of 38 cats were included in this study. Of these, 30 (Group A) were specifically selected for CT evaluation as they tested seropositive for *D. immitis* antibodies and presented with respiratory signs compatible with the differential diagnosis of infection with immature forms of *D. immitis* or HARD (cough, respiratory distress, tachypnea). Additionally, thoracic CT images from 8 cats that were seronegative for *D. immitis* antibodies and asymptomatic (Group B) were also included.

The following inclusion criteria were applied to all feline patients: (i) age > 6 months; (ii) negative result for bronchopulmonary parasites; (iii) absence of cardiovascular or respiratory diseases, or other pathologies that could affect the pulmonary vasculature; and (iv) no medication administered before blood collection. Cats in Group A had to meet the following additional criteria: (v) no prior administration of heartworm preventive treatment.

Blood samples were collected from each animal via the jugular, cephalic or femoral vein, along with identification data (age, sex and breed) and clinical history. Thoracic radiographs in two projections (right lateral and ventrodorsal) were obtained, and hematological and renal parameters were assessed in all patients prior to general anesthesia.

All owners provided informed consent for participation. The cats in Group B underwent CT scans for unrelated clinical conditions (i.e., trauma, neurological disorders), and their thoracic images were included in this study with the owners’ permission. The study was conducted in accordance with current European legislation on animal welfare and protection.

### 2.2. Sample Collection and Assays

Blood samples were collected into serum tubes and centrifuged. The resulting serum was stored at −20 °C until further analysis. To detect IgG antibodies against *D. immitis* in the serum samples, an indirect enzyme-linked immunosorbent assay (ELISA) was performed (in-house ELISA, UranoVet SL, Barcelona, Spain), as previously described [23].

Briefly, each well of the ELISA plate was coated with recombinant *D. immitis* antigens (Di33 protein, 0.5 μg/mL). Serum samples were diluted 1:100 in the sample diluent buffer. After an initial wash to remove unbound components, the conjugate was added to bind with the antigen in each well. Afterwards, a second wash was performed and the substrate (TMB) labeled with horseradish peroxidase was added, which specifically binds to feline IgG. Finally, the reaction was stopped using sulfuric acid to facilitate reading. Optical density was measured at 450 nm. According to the manufacturer’s instructions, samples with a cut-off value ≥ 1 were considered seropositive for *D. immitis* antibodies, while those with values < 1 were classified as seronegative.

Additionally, all serum samples were tested for circulating *D. immitis* antigens using a commercial immunochromatographic test kit (Uranotest Dirofilaria ©, UranoVet SL, Barcelona, Spain) following the manufacturer’s protocol.

### 2.3. CT Scan Image Analysis

CT images were acquired using a helical scanner (Canon Toshiba Astelion, Canon Medical Systems, Tokyo, Japan). Cats were positioned in sternal recumbency with their head extended cranially. A standardized anesthetic protocol was used for all patients, consisting of intravenous midazolam (0.2 mg/kg; Midazolam, B. Braun Medical, Barcelona, Spain) and butorphanol (0.2 mg/kg; Torphadine^®^, Dechra, Northwich, UK) as premedication. Anesthesia was induced with intravenous propofol (0.6 mg/kg; Propofol Lipuro^®^, B. Braun VetCare, Barcelona, Spain), and cats were intubated. Anesthesia was maintained with 2.5% sevoflurane (SevoFlo^®^, Zoetis, Louvain-la-Neuve, Belgium). During the procedure, patients were mechanically ventilated (General Electric, 9100c NXT—SCIL, Boston, MA, USA) and monitored continuously.

An intravenous non-iodinated contrast agent (Xenetix^®^, Guerbet, Roissy, France) was administered at a dose of 600 mgI/kg before image acquisition. The lung window (window level [WL] −500; window width [WW] 1400) was used to visualize and measure the lumens of bronchi, pulmonary arteries, and veins. CT images were obtained and reconstructed at a slice thickness of 1 mm (pitch factor 0.94) using both soft tissue and lung/bone algorithms. The original transverse plane data were used to generate sagittal and dorsal multiplanar reconstructions.

**Figure 1:**
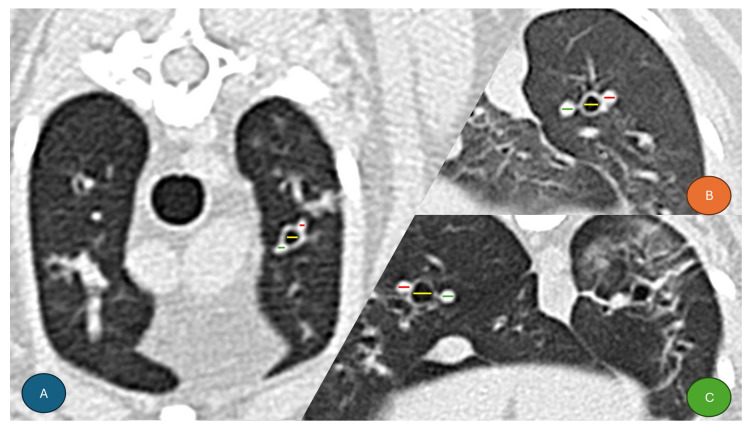
Transverse CT images of the thorax from three seropositive and symptomatic cats with findings compatible with Heartworm-Associated Respiratory Disease (HARD). Colored lines indicate the measurements of the pulmonary artery (red), bronchial lumen (yellow), and pulmonary vein (green), from lateral to medial, respectively. (**A**) Left cranial lobe (cranial subsegment) at T4–T5. (**B**) Left caudal lobe at T9–T10. (**C**) Right caudal lobe at T9–T10.

Measurements were performed in the transverse plane following previously established protocols [20,22], at the following locations: left cranial lobe (cranial subsegment) and right cranial lobe at T4–T5; middle and left cranial lobe (caudal subsegment) at T6–T7; and accessory, left caudal, and right caudal lobes at the T9–T10 intervertebral spaces. Structures were measured transversely (Figure 1). Image analysis was conducted using Horos Version 3 software (LGPL-3.0). All measurements were performed by the same researcher, who was informed of the serological status of each cat before performing the measurements.

### 2.4. Statistical Analysis

Frequencies and percentages were calculated for categorical variables. Differences between groups were evaluated using the nonparametric Pearson’s chi-square test. For 2 × 2 contingency tables, Fisher’s exact test was applied.

For continuous variables, comparisons between groups were performed using the Mann–Whitney U, Kruskal–Wallis, Friedman, or Wilcoxon tests, depending on the specific comparison, due to the small sample size.

Multiple comparisons were adjusted using the Bonferroni correction. All statistical contrasts were accompanied by effect size estimates to aid in the interpretation of results. For continuous variables, the effect size was expressed as r^2^, with the magnitude classified as small (d = 0.1–0.3), medium (d = 0.3–0.5), or large (d > 0.5). For categorical variables, Cramér’s V was used, with the following classification: negligible (0.00–0.09), weak (0.10–0.29), moderate (0.30–0.49), or strong (>0.50).

Different significance levels were used in the analyses (1% [α = 0.01], 5% [α = 0.05], and 10% [α = 0.10]). Statistical analyses were performed using SPSS Base 25.0 software for Windows.

## 3. Results

### 3.1. Descriptive Study

Of the 38 cats included in the study, 16 were male (42.1%) and 22 were female (57.9%). The age of the patients ranged from 1 to 15 years, with a mean of 4.32 years. All animals had similar body weights, with a mean of 3.56 kg.

Group A comprised 30 out of 38 cats (78.95%) that were seropositive for *D. immitis* based on the ELISA technique, while Group B consisted of 8 out of 38 cats (21.05%) that were seronegative. All serum samples (100%) tested negative for circulating *D. immitis* antigens. No statistically significant differences were observed between Groups A and B regarding sex, breed, weight, or age (Table 1).

### 3.2. Comparison of the Ratios According to the ELISA Technique Result

A total of 266 ratio sets (BA, BV, and PV/PA) were obtained from all lung lobes of the cats included in the study. Measurements were carried out by a single researcher (S.N.G.-R.), who has five years of experience in cardiorespiratory medicine and diagnostic imaging. Table 2 summarizes the BA and BV ratios, as well as the diameters of the bronchial lumen, for the two study groups.

In all lung lobes, median BA and BV ratios were significantly higher in Group A cats compared to Group B, except in the accessory lobe, where only the BV ratio differed significantly. Bronchial diameters were also significantly larger in Group A cats in the cranial subsegment of the left cranial lobe, the left caudal lobe, and the right middle lobe. No significant differences between groups were observed for the PV/PA ratio in any lung lobe.

**Figure 2:**
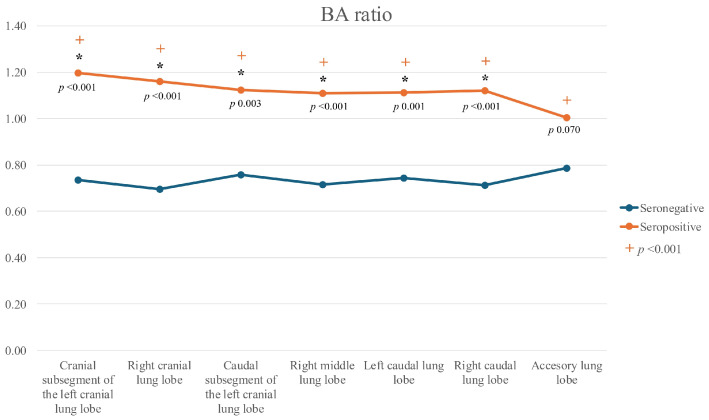
Line graph of bronchoarterial (BA) ratios across all lung lobes in seropositive (Group A) and seronegative (Group B) cats. Legend: (*): significant differences between seropositive and seronegative cats for each lobe; (+): significant differences among lobes within the seropositive group (*p* < 0.001).

**Figure 3:**
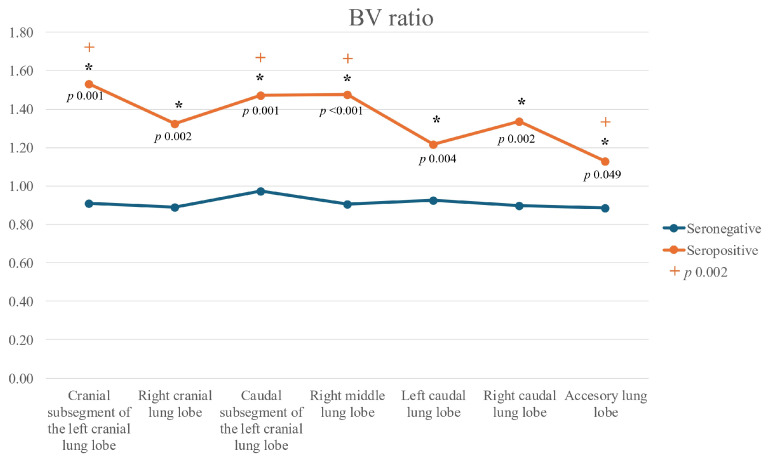
Line graph of bronchus-to-pulmonary vein (BV) ratios across all lung lobes in seropositive (Group A) and seronegative (Group B) cats. Legend: (*): significant differences between seropositive and seronegative cats for each lobe; (+): significant differences among lobes within the seropositive group (*p* = 0.002).

When comparing ratios across different lung lobes within each group, no significant differences were detected in Group B. In contrast, significant differences were observed in Group A for the BA (Friedman(6) = 25.700, *p* < 0.001), BV (Friedman(6) = 20.786, *p* = 0.002), and PV/PA ratios (Friedman(6) = 14.800, *p* = 0.022). For the BA ratio, values in the accessory lobe were significantly lower than in the other lobes (*p* < 0.05) (Figure 2). For the BV ratio, values in the accessory lobe were significantly lower than those in the cranial and caudal subsegments of the left cranial lobe, as well as the right middle lobe (*p* < 0.05) (Figure 3).

**Figure 4:**
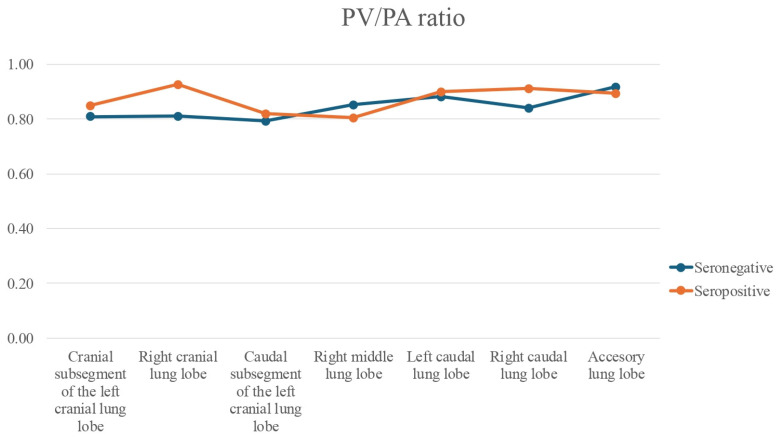
Line graph of pulmonary vein-to-pulmonary artery (PV/PA) ratios across all lung lobes in seropositive (Group A) and seronegative (Group B) cats. No significant differences were found for any lobe, neither between the lung lobes (*p* = 0.022).

However, Bonferroni-corrected pairwise post hoc comparisons did not reveal significant inter-lobe differences for the PV/PA ratio (*p* > 0.05), although the Friedman test indicated a tendency towards variation (Figure 4).

## 4. Discussion

Feline heartworm is a complex condition, with significant pulmonary involvement in both mature and immature forms, and its diagnosis is more challenging than in dogs. Therefore, it requires a multimodal approach that combines serological tests with imaging techniques [2]. In cats infected by *D. immitis*, CT images have shown restrictive lung disease characterized by increased interstitial density and reduced total lung volume, with damage affecting both bronchi and pulmonary arteries [2,24]. CT evaluation of bronchial and vascular structures has thus proven useful for studying and characterizing heartworm in both dogs and cats [18,22,24]. However, in feline infections caused by immature parasites, there are very few imaging studies available. Only one recent report described radiological changes associated with alterations in bronchi, bronchioles, and alveoli in naturally infected cats [25].

In the present study, seropositive cats showed differences in bronchial diameters and in their relation to the pulmonary artery and vein (BA and BV ratios, respectively). These findings suggest that the inflammatory process induced by *D. immitis* larvae and juvenile worms generates early bronchial remodeling, even in immature infections. Bronchi were the most affected structures, with generalized dilation in all lung lobes, reflected by increased BA and BV ratios. This supports the existence of an early bronchial reaction during the initial stages of infection, which corresponds with the symptomatology of HARD. Similar bronchial changes have previously been reported in cats with immature infections by other authors, describing that, histologically, most of the increase in peribronchial wall was muscle mixture of smooth muscle and myofibrosis [2]. Therefore, the present results further reinforce the importance of considering bronchial involvement as a key component of feline heartworm, beyond the vascular compromise typically described in dogs.

The BA ratio, defined by the relation between bronchial lumen and pulmonary artery diameter, has been widely used in CT studies in other species, including dogs [13,26] and humans [18,27,28]. In healthy cats, a mean value of 0.71 ± 0.1 has been reported, while values ≥ 0.91 have been considered abnormal [20]. The present results agree with those of Reid et al. [20], who showed that bronchial structures were more affected than pulmonary arteries, leading to elevated BA ratios. In contrast, other studies evaluating the BA ratio in asthmatic cats did not observe significant differences compared to healthy controls (0.93 ± 0.21 and 0.86 ± 0.12, respectively) [29]. However, these results should be interpreted cautiously given the small sample size (16 healthy and 4 asthmatic cats) and possible breed-related variability.

In experimentally infected cats with *D. immitis*, the bronchial diameters were found to be increased mainly in the right middle and caudal subsegment of the left cranial lobes [22]. By contrast, in this study, dilation of the bronchial lumen was detected in all lung lobes of seropositive cats, with the exception of the accessory lobe, where it was less evident. This discrepancy may be due to differences between experimental and natural infections, including parasite stage (adult vs. immature). Moreover, in the cited experimental study, significant BA ratio differences were restricted to the right cranial and left caudal lobes [22]. The authors concluded that the BA ratio may be less reliable in advanced cases where both pulmonary arteries and bronchi are altered, generating variability in the ratio. In contrast, in the present study the arterial lumens appeared less affected, allowing bronchial changes to predominate and making the BA ratio more sensitive for detecting early involvement. Median BA values in all lobes exceeded 0.91, the upper cut-off suggested to distinguish healthy cats from those with bronchial disease [20]. These findings strengthen the hypothesis that early bronchial reaction is a hallmark of feline heartworm.

The BV ratio has not been previously described in cats. Nevertheless, it may be useful in conditions involving pulmonary venous congestion (e.g., cardiac pathologies, toxicoses, acute inflammation) or bronchial dilation secondary to lower respiratory tract disease. In this study, significant differences in BV ratio were found between seropositive and seronegative cats across all lung lobes. This may be explained by the fact that cats infected with immature *D. immitis* larvae are less prone to venous remodeling than to arterial changes, while bronchial structures are more reactive. Evaluating the BV ratio could therefore provide a benchmark for future studies on lower respiratory tract disease and venous congestion, especially in early or subclinical stages. In feline heartworm, this parameter may be particularly useful for assessing bronchial changes, as venous structures are not typically affected in this disease, unlike arterial structures.

Regarding the PV/PA ratio, no significant differences were observed between groups or among lung lobes. This likely reflects that pulmonary arteries in seropositive cats were not yet significantly altered compared to seronegative cats, consistent with infections by immature stages. These findings contrast with those of Falcón-Cordón et al. [25], who did observe arterial changes in immature infections by thoracic radiography. Such discrepancies may be due to methodological differences in imaging techniques or differences in the cat populations studied (infection stage, duration, or worm burden). Despite the absence of significant alterations in the PV/PA ratio, this index remains clinically relevant due to its utility in diagnosing vascular abnormalities such as pulmonary hypertension and venous congestion [19,30,31,32]. Establishing baseline PV/PA values in naturally infected cats may facilitate the differentiation of heartworm from other cardiopulmonary disorders in endemic areas.

Therefore, the findings of this study indicate that, in clinical practice, assessing BA and BV ratios on CT can help veterinarians in endemic regions to identify early bronchial changes even when serological tests are inconclusive. These quantitative parameters provide objective, reproducible metrics that can strengthen the presumptive diagnosis of feline heartworm and guide appropriate preventive or therapeutic decisions.

This study is the first to describe BA, BV, and PV/PA ratios in cats naturally infected with *D. immitis* across all lung lobes. However, it has several limitations. The small sample size reduces the strength of conclusions. Moreover, the duration of exposure to the parasite and worm burden were unknown, and seropositive cats could have been at different infection stages, leading to heterogeneous findings. Antibody positivity does not distinguish between active and past infections; therefore, some cats may have had HARD, others patent heartworm disease, and others recent infections. Additionally, anesthesia and the respiratory phase during image acquisition can influence bronchial and vascular measurements, as ratios obtained using bronchial measurements at the end of expiration are smaller to those obtained in inspiration and pulmonary vessels can also be influenced by the respiratory cycle where images are acquired [33,34]. In this study, it was not possible to standardize image acquisition using a ventilator. Finally, the fact the researcher was aware of the serological status of each cat before the measurements were taken could have introduced potential bias.

Despite these limitations, the results highlight the relevance of assessing bronchial and vascular alterations in naturally infected cats. Considering the diagnostic challenges of feline heartworm, the inclusion of quantitative imaging parameters may improve suspicion and presumptive diagnosis in endemic regions. Importantly, the prominent bronchial involvement observed emphasizes a differential feature of feline heartworm compared with the canine disease. Nevertheless, further studies with larger populations are needed to confirm and expand upon these results.

## 5. Conclusions

Computed tomography proved valuable for characterizing early pulmonary changes in cats naturally infected with immature *D. immitis*. Bronchial structures were the most affected, with significantly increased BA and BV ratios, while vascular involvement remained limited. These quantitative CT parameters may serve as sensitive and complementary diagnostic tools to conventional serological and imaging methods, supporting early detection and clinical decision-making in endemic areas.

## Figures and Tables

**Table 1:** Data from the 38 cats included in the study, comprising seronegative asymptomatic cats and cats with clinical signs compatible with Heartworm-Associated Respiratory Disease (HARD). Chi-square test (χ^2^) with *p*-values and Cramer’s V were used for qualitative variables (sex, breed), while standardized statistics and Mann–Whitney *p*-values were used for quantitative variables (body weight and age).

		Result of the ELISA Technique			Chi^2^ and *p*-Value	Cramer’s V
		Total (*N*%)	Seronegative (*N*%)	Seropositive (*N*%)		
SEX	Total	38 (100.0%)	8 (100.0%)	30 (100.0%)	Chi^2^(1) = 0.088, *p =* 0.767	1.000
	Female	22	5	17		
	Male	16	3	13		
BREED	Total	38 (100.0%)	8 (100.0%)	30 (100.0%)	Chi^2^(3) = 4.453, *p* = 0.217	
	Common European	33	6	27		
	Persian	1	1	0		
	Siamese	1	0	1		
	Sphynx	3	1	2		
		Result of the ELISA Technique			Standardized Statistical and Mann–Whitney *p*-Value	
		Total (*N*%)	Seronegative (*N*%)	Seropositive (*N*%)		
BODY WEIGHT (kg)	Valid *N*	38	8	30	U = 1.272, *p* = 0.208	
	Median	3.56	3.27	3.95		
	Percentile 25	3.00	2.80	3.00		
	Percentile 75	4.80	3.93	5.00		
AGE	Valid *N*	38	8	30	U = 0.859, *p* = 0.407	
	Median	4.32	3.56	4.68		
	Percentile 25	2.56	1.64	2.66		
	Percentile 75	8.36	6.36	9.62		

**Table 2:** Results of the relationships between bronchial lumen and pulmonary artery (BA), bronchial lumen and pulmonary vein (BV), and bronchial diameters. Legend: (*) *p* < 0.1 (significant at the 90% level); (**) *p* < 0.05 (significant at the 95% level); (***) *p* < 0.01 (significant at the 99% level).

Lung Lobe	Variable	Median (Group A) [RI]	Median (Group B) [RI]	U-Value	*p*-Value	Effect Size (r)	Magnitude
Left cranial (cranial subsegment)	BA	1.20 [0.96–1.48]	0.73 [0.65–0.79]	3.795	<0.001 ***	>0.5	Large
	BV	1.53 [1.03–1.90]	0.91 [0.81–1.04]	3.115	0.001 ***	>0.5	Large
	Bronchus	1.25 [1.11–1.71]	0.91 [0.83–1.26]	2.148	0.031 **	0.3–0.5	Medium
Left cranial (caudal subsegment)	BA	0.99 [0.79–1.48]	0.76 [0.70–0.83]	2.864	0.003 ***	0.3–0.5	Medium
	BV	1.47 [1.16–1.69]	0.97 [0.89–1.09]	3.043	0.001 ***	0.3–0.5	Medium
	Bronchus	1.21 [1.02–1.71]	1.02 [0.71–1.62]	1.021	0.314		
Left caudal	BA	1.11 [0.86–1.33]	0.74 [0.71–0.81]	3.079	0.001 ***	0.3–0.5	Medium
	BV	1.22 [1.06–1.67]	0.92 [0.83–0.99]	2.757	0.004 ***	0.3–0.5	Medium
	Bronchus	2.32 [1.80–2.75]	1.81 [1.63–1.92]	2.077	0.038 **	0.3–0.5	Medium
Right cranial	BA	1.16 [0.98–1.55]	0.70 [0.65–0.77]	3.294	<0.001 ***	>0.5	Large
	BV	1.33 [1.05–1.62]	0.89 [0.74–1.07]	2.936	0.002 ***	0.3–0.5	Medium
	Bronchus	1.39 [0.98–1.72]	0.96 [0.80–1.32]	1.647	0.104		
Right middle	BA	1.11 [0.94–1.47]	0.72 [0.67–0.77]	3.760	<0.001 ***	>0.5	Large
	BV	1.48 [1.08–1.85]	0.91 [0.80–1.02]	3.545	<0.001 ***	>0.5	Large
	Bronchus	1.10 [0.91–1.41]	0.71 [0.61–0.84]	2.524	0.010 **	0.3–0.5	Medium
Right caudal	BA	1.12 [1.01–1.38]	0.71 [0.62–0.79]	3.330	<0.001 ***	>0.5	Large
	BV	1.34 [1.07–1.53]	0.90 [0.70–0.96]	3.008	0.002 ***	>0.5	Large
	Bronchus	2.49 [2.93–1.90]	2.03 [2.54–1.46]	1.647	0.104		
Accessory	BA	1.00 [0.85–1.17]	0.79 [0.68–1.04]	1.826	0.070 *		
	BV	1.13 [0.91–1.32]	0.88 [0.79–0.96]	1.969	0.049 **	0.3–0.5	Medium
	Bronchus	0.89 [0–75–1.15]	0.82 [0.70–0.97]	0.895	0.388		

## Data Availability

The raw data supporting the conclusions of this article will be made available by the authors, without undue reservation.
